# Low expression of microRNA-328 can predict sepsis and alleviate sepsis-induced cardiac dysfunction and inflammatory response

**DOI:** 10.1590/1414-431X20209501

**Published:** 2020-06-19

**Authors:** Bin Sun, Chunye Luan, Lisha Guo, Bing Zhang, Yufang Liu

**Affiliations:** 1 Department of Emergency, Binzhou Medical University Hospital, Binzhou, Shandong, China; 2 Department of Gynaecology and Obstetrics, Binzhou Medical University Hospital, Binzhou, Shandong, China

**Keywords:** miR-328, Sepsis, Cardiac dysfunction, Inflammatory response, Diagnosis

## Abstract

Sepsis often leads to cardiac dysfunction and inflammation. This study investigated the clinical value of microRNA-328 (miR-328) in sepsis and its role in cardiac dysfunction and inflammation caused by sepsis. The expression level of miR-328 in the serum of the subjects was detected by qRT-PCR. Receiver operating characteristic (ROC) curve measured the diagnostic value of miR-328 in sepsis. Rat sepsis model was established to detect left ventricular systolic pressure (LVSP), left ventricular end-diastolic pressure (LVEDP), and maximal rate of increase/decrease of left ventricular pressure (±dp/dt_max_). Myocardial injury markers serum cardiac troponin I (cTnI), myocardial kinase isoenzyme (CK-MB), and inflammatory factors were detected by enzyme-linked immunosorbent assay (ELISA). miR-328 expression was assessed in serum of sepsis patients and in rat models of sepsis. The AUC of ROC curve was 0.926, sensitivity 87.60%, and specificity 86.36%. Compared with the sham group, LVSP and +dp/dt_max_ were decreased in the rat model of sepsis. LVEDP, -dp/dt_max_, cTnI, CK-MB, tumor necrosis factor-α, interleukin (IL)-6, and IL-1β were upregulated in the rat model of sepsis. The low expression of miR-328 reversed these indicators. miR-328 is a diagnostic marker for patients with sepsis, and decreasing the expression level of miR-328 can ameliorate cardiac dysfunction and cardiac inflammation in sepsis.

## Introduction

Sepsis has been identified as a systemic inflammatory response to infection or injury (1). About 50% of sepsis patients require intensive care unit treatment (2,3), leading to high morbidity and mortality (4,5). The annual incidence of severe sepsis is increasing, with a recent report showing 132 cases of patients per 100,000 people, a mortality rate of nearly 50%, and the high financial burden of $50,000 per patient for nursing care (6,7). Cardiac dysfunction is one of the common complications of sepsis-induced death (8,9). Myocardial injury and myocardial depression are the most common cardiac dysfunctions caused by sepsis (10). In the diagnosis of sepsis, the analysis of blood microbial culture is the gold standard, but it takes a long time and an early positive rate is low (11). As a systemic inflammatory response, inflammation-related serum C-reactive protein (CRP), procalcitonin (PTC), and interleukin (IL-6) have been used in the diagnosis of sepsis (12,13), but their specificity and sensitivity are limited by different conditions. Therefore, new biomarkers are urgently needed for the early diagnosis and accurate assessment of sepsis patients.

MicroRNA are endogenous, non-coding small RNA molecules consisting of 22 nucleotides. Abnormal expression of miRNA has been detected in a variety of human disease states including sepsis, cardiovascular disease, inflammation, and tumor. miR-328 is a miRNA located on chromosome 16q22.1 and is involved in various diseases including lung cancer (14), osteosarcoma (15), nasopharyngeal (16), myocardial infarction (17), and chronic leukemia (18). A recent study indicated that miR-328 can promote myocardial fibrosis through paracrine regulation of cardiomyocytes (19), resulting in cardiac dysfunction. Additionally, miR-328 promotes cardiac fibrosis by stimulating the tumor growth factor-β1 signaling pathway to promote collagen production (20). All evidence suggests a crucial role for miR-328 in myocardial function. Furthermore, miR-328 is also reported to be significantly upregulated in Kawasaki disease with acute systemic vasculitis (21), indicating its important role in inflammatory response. However, the role of miR-328 in sepsis and sepsis-induced cardiac dysfunction and inflammation is unknown.

This study investigated the expression level and clinical role of miR-328 in patients with sepsis. In addition, the role of miR-328 in cardiac dysfunction and inflammation caused by sepsis was studied.

## Material and Methods

### Subject data and blood samples

Patients with sepsis who were admitted to the intensive care unit of Binzhou Medical University Hospital from January 2016 to December 2017 were recruited. The inclusion criteria for this study were based on consensus definition of the American College of Chest Physicians/Intensive Care Medicine, and the results of blood microbial culture were used to diagnose sepsis in patients (22). Patients with the following conditions were excluded: 1) receiving immunosuppressive drugs within the last 3 months; 2) pregnancy or lactation; 3) severe chronic diseases such as heart, liver, kidney, and lung; 4) suffering from solid cancer or hematological malignancies; 5) human immunodeficiency virus infection; and 6) immunodeficiency. A total of 110 patients were studied after screening. At the same time, 89 healthy volunteers of similar age and gender and no systemic inflammation, tumor history, or heart and kidney dysfunction were recruited from the health check-up center. Organ dysfunction (SOFA) score (23) and acute physiology and chronic health (APACHE-II) score (24 [Bibr B25] [Bibr B26]) were applied in patients with sepsis within 24 h of initial admission to the ICU, and blood samples were collected from patients for testing. Clinical information such as age, gender, body mass index (BMI), serum creatinine (Scr), white blood cells (WBC), albumin, PTC, and CRP were recorded.

The study was approved by the Medical Ethics Committee of Binzhou Medical University Hospital and all participants or family members signed the consent form. All patients were treated according to current guidelines for the treatment of sepsis (survival of sepsis) and specific guidelines from relevant committees.

### Animals and animal model of sepsis

Forty adult male Sprague-Dawley rats weighing 250-300 g were purchased from Shanghai Animal Center (China). Rats were housed at 22–23°C, 50% humidity, with a 12/12 light and dark schedule for at least 5 days for adaptation before conducting experiments. All animal experiments were performed in accordance with the animal care and use guidelines of the Binzhou Medical University Hospital Ethics Committee. Establishment of the model of cecal ligation and perforation (CLP) sepsis in rats was done in a sterile environment. First, the rats were anesthetized with 50 mg/kg pentobarbital sodium and then treated with 75% ethanol for intraperitoneal disinfection. After anesthesia, the rats were incised 2 cm in the middle of the lower abdomen to expose the cecum, which was ligated. A sterile needle 18 was used to puncture the cecum twice, and the cecum was put back into the abdominal cavity after gently squeezing out the feces. Finally the abdominal incision was sutured. The sham group had the same operation except with no ligation and puncture. After the operation, the rats were placed on a constant temperature heating pad, and the body temperature was maintained at 36–37°C.

### Groups of animals

Rats were divided into 4 groups with 10 rats in each group: sham group, CLP group, miR-328 negative control group (miR-328 NC), and miR-328 antagomir group. In the sham group, 1 mL of normal saline was injected into the tail vein 24 h before surgery. In the CLP group, 1 mL of normal saline was injected into the tail vein of CLP-treated rats 24 h before surgery. The miR-328 NC group was injected with 10 μg miR NC sequence into the tail vein of CLP-treated rats 24 h before surgery. The miR-328 antagomir group was injected with 10 μg miR antagomir sequence into the tail vein of CLP-treated rats 24 h before surgery. After successful modeling, at least 8 individuals survived in each group. miR-328 NC and miR-328 antagomir were synthesized by GenePharma (China).

### Cardiac function measurement and blood cytokines assessments

Rats were tested for cardiac function after surgery. After the rats were anesthetized, a catheter was inserted into the left ventricle through the right common artery. Analyses of changes in rat hemodynamic parameters, such as left ventricular end-diastolic pressure (LVEDP), left ventricular systolic pressure (LVSP), the maximum rate of increase in left ventricular blood pressure (+ dp/dt_max_), and the maximum rate of decrease in left ventricular blood pressure (- dp/dt_max_), were performed using the MFLab 3.01 software in the FDP-1 HRV & BRS analysis system. After cardiac function was measured, 5 mL of inferior vena cava blood was placed into a test tube with added anticoagulant, and serum was collected by centrifugation at 2,500 *g* for 15 min at room temperature for enzyme-linked immunoreactivity assay (ELISA). ELISA kit (Abcam, UK) was used for the detection of tumor necrosis factor α (TNF-α), interleukin 1β (IL-1β), interleukin 6 (IL-6), and biomarkers of myocardial injury in rat serum. Expression levels of creatine kinase isoenzyme (CK-MB) and troponin (cTnI) were read at 450 nm absorbance.

### Quantitative real-time polymerase chain reaction (qRT-PCR)

After the end of the rat experiment, each group of rats was sacrificed by cervical dislocation. Total RNA in heart tissue was isolated using TRIZOL. RNA extraction reagent (Invitrogen, USA) was used, and total RNA reverse transcription was performed using the PrimerScript Real-time reagent kit (TAKARA, Japan). Finally, qRT-PCR was detected by ABI PRISM 7000 (Applied Biosystems, USA) by SYBR Premix Ex Taq TM II reagent. Using U6 as an internal reference, the relative expression of miR-328 was calculated by 2^−ΔΔCt^.

### Statistical analysis

Data are reported as means±SD, and Student's *t*-test was used to compare differences between two groups. The receiver operating characteristic (ROC) curve was drawn to evaluate the diagnostic value of miR-328 level in patients with sepsis, and the area under the curve (AUC) was calculated. Spearman correlation analysis examined the relationship between miR-328 expression and clinical characteristics of patients. Statistical analysis was performed using SPSS 19.0 software and GraphPad Prism 7.0 software (USA). P<0.05 was considered to be statistically significant.

## Results

### Clinical characteristics of study subjects

One hundred and ten sepsis patients (69 males/41 females, mean age 54.85±4.99) and 89 healthy controls (58 males/31 females, mean age 55.88±4.90) were enrolled. The demographic and clinical characteristics of the two groups of subjects are shown in Table 1. There was no significant difference in age (P<0.150), gender (P<0.768), and BMI (P<0.258) between the healthy control group and the sepsis patients group, but significant differences in Scr, WBC, CRP, and PTC were observed (P<0.001). At the same time, APACHE II (12.09±2.30) and SOFA (4.8±1.25) scores of sepsis patients were significantly higher than those of the healthy control group.

**Table 1:** Comparison of the baseline data between the healthy control group and the sepsis group of patients.

Parameters	Healthy (n=89)	Sepsis (n=110)	P value
Age (years)	55.88±4.91	54.85±4.99	0.150
Gender (male/female)	58/31	69/41	0.723*
BMI (kg/m^2^)	20.83±1.19	20.63±1.21	0.258
Scr (mg/dL)	0.97±0.21	1.61±0.188	<0.001
Albumin (g/L)	40.76±3.07	30.11±5.50	<0.001
WBC (×10^9^/L)	7.48±1.56	18.43±3.19	<0.001
CRP (mg/L)	7.10±2.07	69.12±15.07	<0.001
PCT (ng/mL)	0.06±0.02	11.54±2.62	<0.001
APACHE II score	-	12.09±2.30	-
SOFA score	-	4.8±1.25	-

BMI: body mass index; Scr: serum creatinine; WBC: white blood cells; CRP: C-reactive protein; PCT: procalcitonin; APACHE: acute physiology and chronic health evaluation; SOFA: sequential organ failure assessment. Data are reported as means±SD (Student's *t*-test or *chi-squared test).

### Serum level of miR-328 in sepsis patients

**Figure 1:**
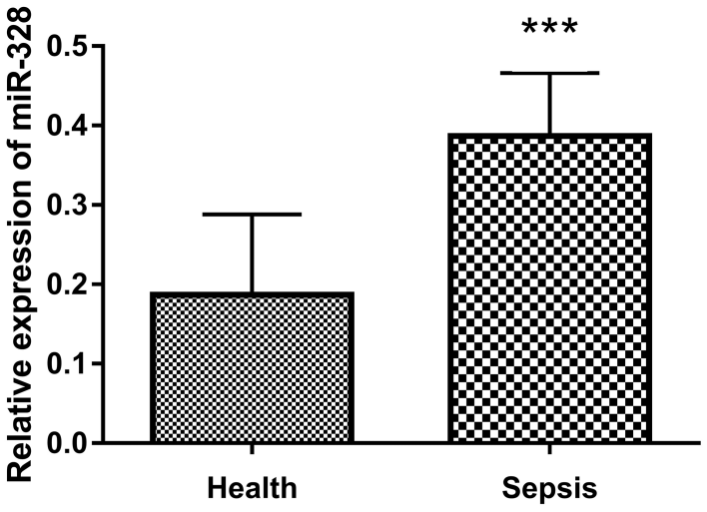
The expression level of miR-328 in the serum of sepsis patients and healthy controls was detected by qQT-PCR. Data are reported as means±SD. ***P<0.001 (Student's *t*-test).

The expression level of miR-328 in patients with sepsis was significantly higher than that in the healthy control group (P<0.001). Therefore, it was speculated that miR-328 played a crucial role in sepsis (Figure 1).

### Correlation of miR-328 expression with clinicopathological features of sepsis patients

The expression of miR-328 was significantly positively correlated with Scr, WBC, CRP, PTC, APACHE II score, and SOFA score (P<0.01), but had no significant correlation with age, gender, BMI, and albumin (P>0.05). The experimental results showed that the expression level of miR-328 was significantly positively correlated with the condition of sepsis (Table 2).

**Table 2:** Correlation of miR-328 relative expression with clinical characteristics of sepsis patients.

Parameters	MiR-328 expression	
	P value	Correlation coefficient (r)
Age	0.223	-0.117
Gender	0.665	-0.042
BMI	0.253	-0.110
Scr	0.009	0.248
Albumin	0.099	-0.158
WBC	<0.001	0.395
CRP	<0.001	0.486
PCT	<0.001	0.425
APACHE II score	<0.001	0.577
SOFA score	<0.001	0.552

BMI: body mass index; Scr: serum creatinine; WBC: white blood cell; CRP: C-reactive protein; PCT: procalcitonin; APACHE: acute physiology and chronic health evaluation; SOFA: sequential organ failure assessment. Spearman correlation analysis.

### Diagnostic value of miR-328 in sepsis

**Figure 2:**
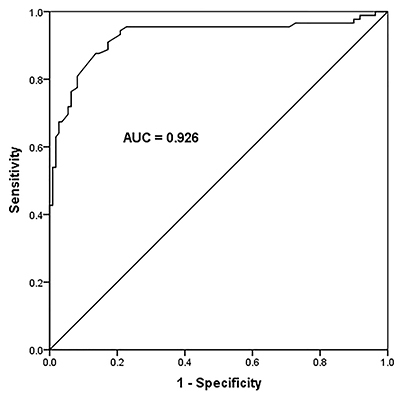
The receiver operating characteristic curve was used to analyze the diagnostic value of miR-328 in sepsis. The area under the curve (AUC) was 0.926, the sensitivity was 87.60%, and the specificity was 86.36%.

The AUC of miR-328 was 0.926, the cut-off value was 0.305, the sensitivity was 87.60%, and the specificity was 86.36%. The ROC curve showed that miR-328 has a good diagnostic value for sepsis (Figure 2).

### Effect of miR-328 on cardiac dysfunction in sepsis rat model

**Figure 3:**
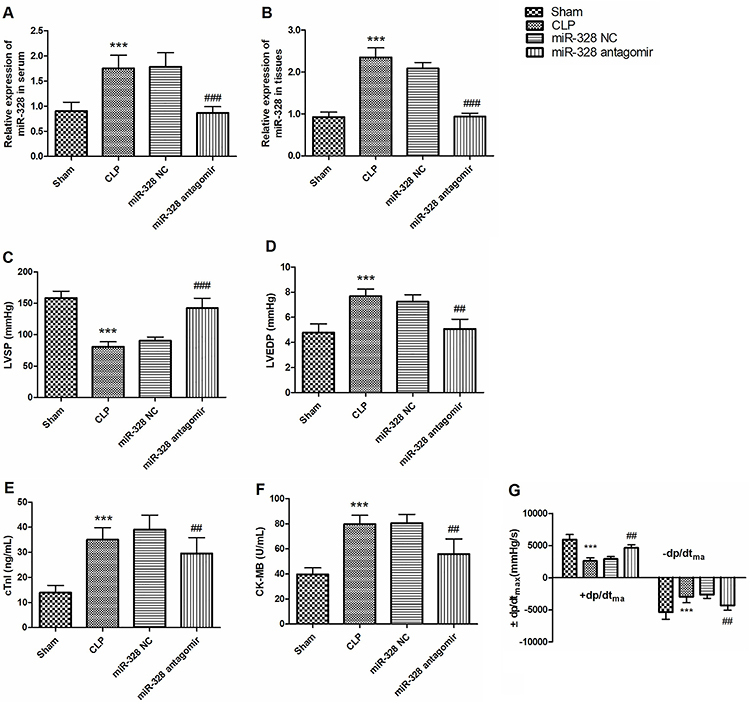
Effect of miR-328 on cardiac dysfunction in a rat model of sepsis. **A** and **B**, Changes in expression levels of miR-328 in serum and myocardial tissue after establishment of a rat model of sepsis and after injection of miR-328 antagomir. **C**-**G**, Modeling of sepsis in rats and changes in cardiac hemodynamics and serum myocardial injury after miR-328 antagomir injection. Data are reported as means±SD. ***P<0.001, compared with sham group; ^##^P<0.01, ^###^P<0.001, compared with CLP group (Student's *t*-test). CLP: cecal ligation and perforation; NC: negative control; LVSP: left ventricular systolic pressure; LVDEP: left ventricular end-diastolic pressure; cTnI: serum cardiac troponin I; CK-MB: myocardial kinase isoenzyme; ±dp/dt_max_: maximum rate of increase/decrease in left ventricular blood pressure.

The expression of miR-328 was significantly up-regulated in rat tissues and serum after CLP modeling, but the high expression was reversed when miR-328 antagomir was injected (P<0.01, Figure 3A and B). In addition, compared with the sham group, LVSP and +dp/dt_max_ decreased significantly in the CLP group, while the levels of -dp/dt_max_, LVEDP, cTnI, and CK-MB were significantly increased (P<0.01, Figure 3C–G). The experimental results showed that myocardial dysfunction occurred in the rat model of sepsis. However, when miR-328 antagomir was injected, myocardial dysfunction in sepsis rats was reversed, LVSP and +dp/dt_max_ were significantly increased, levels of -dp/dt_max_, LVEDP, cTnI, and CK-MB were significantly decreased (P<0.001, Figure 3C-G). Therefore, it was determined by the rat sepsis model that miR-328 may be a potential mechanism for the regulation of myocardial function in sepsis.

### Effect of miR-328 on inflammatory responses in sepsis rat model

**Figure 4:**
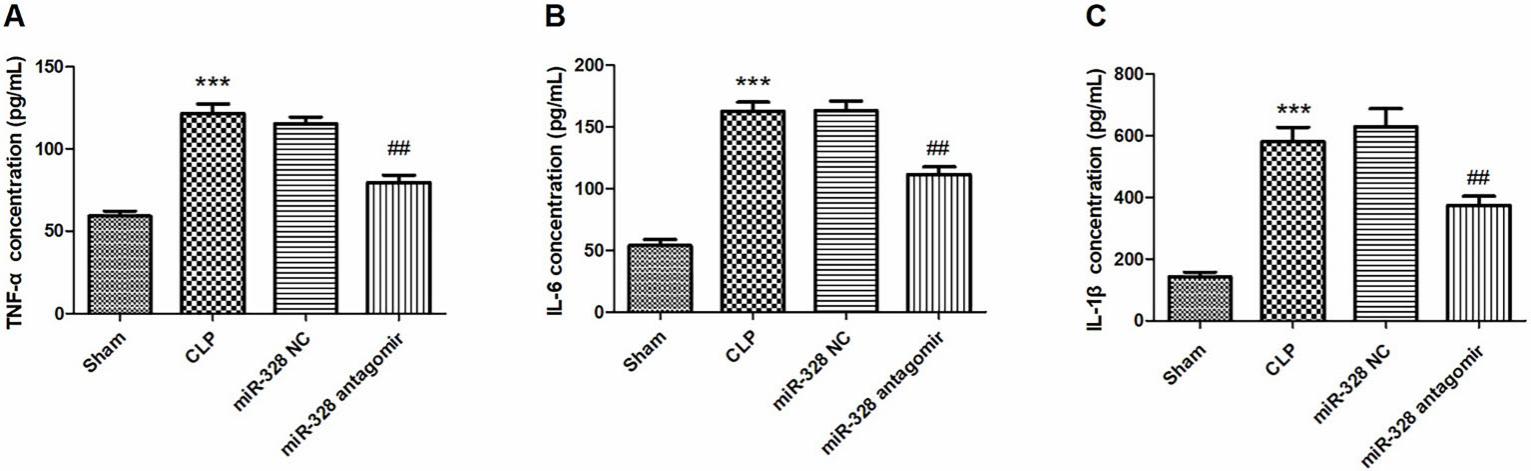
Changes in inflammatory factors in the sepsis rat model by cecal ligation and perforation (CLP) and after injection of miR-328 antagomir. Data are reported as means±SD. ***P<0.001, compared with sham group; ^##^P<0.01, compared with CLP group (Student's *t*-test). NC: negative control. TNF-α: tumor necrosis factor-α; IL: interleukin.

The expression levels of TNF-α, IL-6, and IL-1β were significantly increased in the CLP group compared with the sham group, indicating that sepsis promoted inflammation. However, injection of miR-328 antagomir reduced the inflammatory response, resulting in decreased levels of TNF-α, IL-6, and IL-1β expression (P<0.01, Figure 4A–C). The results of this study demonstrated that miR-328 affects changes in the inflammatory response in sepsis.

## Discussion

Sepsis is a common cause of death in hospitalized patients worldwide and is still a huge challenge in clinical medicine due to its high mortality and morbidity. The current diagnostic markers CRP, IL-6, PTC, and other biomarkers are more sensitive but less specific, so new diagnostic markers must be investigated to help determine the most appropriate treatment to reduce patient mortality (25,26).

miRNAs are part of a complex regulatory network in the regulation of physiological and pathological processes of gene expression. Abnormal expression of miRNA is not only confirmed in development, aging, and cell death (27,28), but is also found in complex diseases such as infection, inflammation, and sepsis (29–31). Previous studies have reported that miR-328 is abnormally expressed in a variety of diseases and can be used as a marker for diagnosis and prognosis. miR-328 is significantly down-regulated in gliomas and can inhibit cell proliferation and invasion, being a good prognostic marker for glioma (32). High expression of miR-328 in peripheral blood is an effective marker for early diagnosis of non-small cell lung cancer (33). In our study, we have demonstrated for the first time that miR-328 was highly expressed in patients with sepsis, and the ROC curve confirmed that miR-328 was an effective diagnostic marker for sepsis.

Previous studies have confirmed that cardiac dysfunction as a common complication of sepsis can seriously affect a patient's health and is associated with increased mortality (34). LVSP and +dp/dt_max_ are key indicators of myocardial contractility, and LVEDP and -dp/dt_max_ are valuable indicators of myocardial relaxation (35). In our study, the levels of LVSP and +dp/dt_max_ were significantly reduced in the CLP group, while the levels of LVEDP and -dp/dt_max_ were significantly elevated, indicating that myocardial contraction and diastolic dysfunction were caused by sepsis. cTnI and CK-MB are specific biomarkers for myocardial injury (36). In our study, the levels of cTnI and CK-MB in the CLP group were significantly elevated, and experiments confirmed that sepsis can cause myocardial damage. This was consistent with previous findings that sepsis can lead to cardiac dysfunction. A study by Zhao et al. (19) in 2018 found that cardiomyocyte-derived miR-328 can promote myocardial fibrosis by paracrine regulation of adjacent fibroblasts. In 2016, Du et al. (20) confirmed that miR-328 was up-regulated in the marginal zone of myocardial infarction in mice, confirming that miR-328 is an effective miRNA that promotes fibrosis. In the present study, it was confirmed that down-regulation of miR-328 expression level alleviated cardiac dysfunction caused by sepsis, such as myocardial contractile function inhibition, myocardial diastolic function promotion, and myocardial injury. The experimental results confirmed that in sepsis, the down-regulation of miR-328 helped to alleviate cardiac dysfunction and provided protection for the myocardium.

Sepsis is a systemic inflammatory response syndrome that is the leading cause of cardiac dysfunction (37). We have demonstrated that inhibition of miR-328 can ameliorate cardiac dysfunction in sepsis, and it has been reported that miR-328 is associated with inflammatory response, so we further speculated whether miR-328 can affect the inflammatory response in sepsis. Zhang et al. (21) confirmed that miR-328 in the serum of patients with Kawasaki disease caused by systemic vascular inflammation is significantly up-regulated and can be used as a biomarker for diagnosis and prediction. Meanwhile, recent studies have reported that five miRNAs, including miR-328, play a regulatory role in non-small cell lung cancer by affecting inflammation-related signaling pathways (38). Consistently, in our experiment, inflammatory cytokines were significantly up-regulated in the CLP group, confirming that sepsis can promote the occurrence of inflammatory response. The decrease of miR-328 reduced the expression of inflammatory factors in sepsis.

In conclusion, miR-328 was highly expressed in the serum of patients with sepsis, and down-regulation of miR-328 can alleviate cardiac dysfunction and inflammatory response in sepsis. miR-328 can be used as a diagnostic marker for sepsis for early diagnosis and treatment.

## References

[1] Venet F, Rimmele T, Monneret G (2018). Management of sepsis-induced immunosuppression. Crit Care Clin.

[2] Angus DC, Linde-Zwirble WT, Lidicker J, Clermont G, Carcillo J, Pinsky MR (2001). Epidemiology of severe sepsis in the United States: analysis of
incidence, outcome, and associated costs of care. Crit Care Med.

[3] Lagu T, Rothberg MB, Shieh MS, Pekow PS, Steingrub JS, Lindenauer PK (2012). Hospitalizations, costs, and outcomes of severe sepsis in the
United States 2003 to 2007. Crit Care Med.

[4] Lago AF, de Oliveira AS, de Souza HCD, da Silva JS, Basile-Filho A, Gastaldi AC (2018). The effects of physical therapy with neuromuscular electrical
stimulation in patients with septic shock: study protocol for a randomized
cross-over design. Medicine.

[5] Venkatesh B, Finfer S, Cohen J, Rajbhandari D, Arabi Y, Bellomo R (2018). Adjunctive glucocorticoid therapy in patients with septic
shock. N Engl J Med.

[6] Chalfin DB, Holbein ME, Fein AM, Carlon GC (1993). Cost-effectiveness of monoclonal antibodies to gram-negative
endotoxin in the treatment of gram-negative sepsis in ICU
patients. JAMA.

[7] Dombrovskiy VY, Martin AA, Sunderram J, Paz HL (2007). Rapid increase in hospitalization and mortality rates for severe
sepsis in the United States: a trend analysis from 1993 to
2003. Crit Care Med.

[8] Yende S, Linde-Zwirble W, Mayr F, Weissfeld LA, Reis S, Angus DC (2014). Risk of cardiovascular events in survivors of severe
sepsis. Am J Resp Crit Care Med.

[9] Sato R, Kuriyama A, Takada T, Nasu M, Luthe SK (2016). Prevalence and risk factors of sepsis-induced cardiomyopathy: a
retrospective cohort study. Medicine.

[10] Zanotti-Cavazzoni SL, Hollenberg SM (2009). Cardiac dysfunction in severe sepsis and septic
shock. Curr Opin Crit Care.

[11] Otto GP, Sossdorf M, Claus RA, Rodel J, Menge K, Reinhart K (2011). The late phase of sepsis is characterized by an increased
microbiological burden and death rate. Crit Care.

[12] Pierrakos C, Vincent JL (2010). Sepsis biomarkers: a review. Crit Care.

[13] Bloos F, Reinhart K (2014). Rapid diagnosis of sepsis. Virulence.

[14] Shen M, Cai L, Jiang K, Xu W, Chen Y, Xu Z (2018). The therapeutic role of inhibition of miR-328 on pulmonary
carcinoma induced by chlamydia pneumoniae through targeting histone
H2AX. Cancer Biomark.

[15] Zhang M, Zhang J, Zhou Q (2019). Elevated expression of microRNA-328-3p suppresses aggressive
malignant behaviors via targeting matrix metalloprotease 16 in
osteosarcoma. Onco Targets Ther.

[16] Lin CH, Chiang MC, Chen YJ (2018). MicroRNA-328 inhibits migration and epithelial-mesenchymal
transition by targeting CD44 in nasopharyngeal carcinoma
cells. Onco Targets Ther.

[17] He F, Lv P, Zhao X, Wang X, Ma X, Meng W (2014). Predictive value of circulating miR-328 and miR-134 for acute
myocardial infarction. Mol Cell Biochem.

[18] Eiring AM, Harb JG, Neviani P, Garton C, Oaks JJ, Spizzo R (2010). miR-328 functions as an RNA decoy to modulate hnRNP E2 regulation
of mRNA translation in leukemic blasts. Cell.

[19] Zhao D, Li C, Yan H, Li T, Qian M, Zheng N (2018). Cardiomyocyte derived miR-328 Promotes cardiac fibrosis by
paracrinely regulating adjacent fibroblasts. Cell Physiol Biochem.

[20] Du W, Liang H, Gao X, Li X, Zhang Y, Pan Z (2016). MicroRNA-328, a potential anti-fibrotic target in cardiac
interstitial fibrosis. Cell Physiol Biochem.

[21] Zhang X, Xin G, Sun D (2018). Serum exosomal miR-328, miR-575, miR-134 and miR-671-5p as
potential biomarkers for the diagnosis of Kawasaki disease and the
prediction of therapeutic outcomes of intravenous immunoglobulin
therapy. Exp Ther Med.

[22] Bone RC, Balk RA, Cerra FB, Dellinger RP, Fein AM, Knaus WA (1992). Definitions for sepsis and organ failure and guidelines for the
use of innovative therapies in sepsis. The ACCP/SCCM Consensus Conference
Committee. American College of Chest Physicians/Society of Critical Care
Medicine. Chest.

[23] Vincent JL, Moreno R, Takala J, Willatts S, De Mendonca A, Bruining H (1996). The SOFA (Sepsis-related Organ Failure Assessment) score to
describe organ dysfunction/failure. On behalf of the Working Group on
Sepsis-Related Problems of the European Society of Intensive Care
Medicine. Intensive Care Med.

[24] Knaus WA, Draper EA, Wagner DP, Zimmerman JE (1985). APACHE II: a severity of disease classification
system. Crit Care Med.

[B25] Dhas BB, Dirisala VR, Bhat BV (2018). Expression levels of candidate circulating microRNAs in
early-onset neonatal sepsis compared with healthy newborns. Genomics Insights.

[B26] Fabri-Faja N, Calvo-Lozano O, Dey P, Terborg RA, Estevez MC, Belushkin A (2019). Early sepsis diagnosis via protein and miRNA biomarkers using a
novel point-of-care photonic biosensor. Anal Chim Acta.

[27] Xia HL, Lv Y, Xu CW, Fu MC, Zhang T, Yan XM (2017). MiR-513c suppresses neuroblastoma cell migration, invasion, and
proliferation through direct targeting glutaminase (GLS). Cancer Biomark.

[28] Huang F, Wang B, Zeng J, Sang S, Lei J, Lu Y (2018). MicroRNA-374b inhibits liver cancer progression via down
regulating programmed cell death-1 expression on cytokine-induced killer
cells. Oncol Lett.

[29] Benz F, Roy S, Trautwein C, Roderburg C, Luedde T (2016). Circulating MicroRNAs as biomarkers for sepsis. Int J Mol Sci.

[B30] Liu Y, Cheng Z, Pan F, Yan W (2017). MicroRNA-373 promotes growth and cellular invasion in
osteosarcoma cells by activation of the PI3K/AKT-Rac1-JNK pathway: the
potential role in spinal osteosarcoma. Oncol Res.

[31] Zhang X, Ai F, Li X, Tian L, Wang X, Shen S (2017). MicroRNA-34a suppresses colorectal cancer metastasis by
regulating notch signaling. Oncol Lett.

[32] Yuan J, Zheng Z, Zheng Y, Lu X, Xu L, Lin L (2016). microRNA-328 is a favorable prognostic marker in human glioma via
suppressing invasive and proliferative phenotypes of malignant
cells. Int J Neurosci.

[33] Ulivi P, Foschi G, Mengozzi M, Scarpi E, Silvestrini R, Amadori D (2013). Peripheral blood miR-328 expression as a potential biomarker for
the early diagnosis of NSCLC. Int J Mol Sci.

[34] Lv X, Wang H (2016). Pathophysiology of sepsis-induced myocardial
dysfunction. Mil Med Res.

[35] Chen H, Wang X, Yan X, Cheng X, He X, Zheng W (2018). LncRNA MALAT1 regulates sepsis-induced cardiac inflammation and
dysfunction via interaction with miR-125b and p38
MAPK/NFkappaB. Int Immunopharmacol.

[36] Chen S, Hua F, Lu J, Jiang Y, Tang Y, Tao L (2015). Effect of dexmedetomidine on myocardial ischemia-reperfusion
injury. Int J Clin Exp Med.

[37] Pathan N, Franklin JL, Eleftherohorinou H, Wright VJ, Hemingway CA, Waddell SJ (2011). Myocardial depressant effects of interleukin 6 in meningococcal
sepsis are regulated by p38 mitogen-activated protein kinase. Crit Care Med.

[38] Zhang Y, Roth JA, Yu H, Ye Y, Xie K, Zhao H (2019). A 5-microRNA signature identified from serum microRNA profiling
predicts survival in patients with advanced stage non-small cell lung
cancer. Carcinogenesis.

